# A Systematic Review of Current Terminology for Conditions Preceding Degenerative Cervical Myelopathy: Evidence Synthesis to Inform an AO Spine Expert Opinion Statement

**DOI:** 10.1177/21925682251339480

**Published:** 2025-04-30

**Authors:** Vinisha Agrawal, Mohammed Farhan Ali, Froher Yasin, Daniyal Ashraf, Jamie FM Brannigan, Ratko Yurac, Vishal Kumar, Rory Murphy, Enrico Tessitore, Granit Molliqaj, Joost Dejaegher, Juan José Zamorano, Guy Wynne-Jones, Manjul Tripathi, David B Anderson, Jose Joefrey F Arbatin, So Kato, Ronie Romelean Jayapalan, Nicolas Dea, James S. Harrop, Jefferson Wilson, Brian K. Kwon, Allan R. Martin, Josef Bednarik, Mark R Kotter, Benjamin M Davies, Oliver D Mowforth, Aria Nouri

**Affiliations:** 1 School of Clinical Medicine, 2152 University of Cambridge, Cambridge, UK; 2 Division of Orthopaedic Surgery, Clinica Alemana de Santiago, Universidad del Desarrollo, Santiago, Chile; 3 Division of Orthopaedic Surgery, 29751 PGIMER, Chandigarh, India; 4 Department of Neurosurgery, 115467 Barrow Neurological Institute, Phoenix, AZ, United States; 5 Division of Neurosurgery, Geneva University Hospitals, 27212 University of Geneva, Geneva, Switzerland; 6 Department of Neurosurgery, Faculty of Medicine KU Leuven, Leuven, Belgium; 7 Division of Orthopaedic Surgery, Hospital del Trabajador, Santiago, Chile; 8 Department of Neurosurgery, The Newcastle Upon Tyne Hospitals NHS Foundation Trust, Newcastle, UK; 9 Department of Neurosurgery, 29751 PGIMER, Chandigarh, India; 10 School of Health Sciences, Faculty of Medicine and Health, University of Sydney, Sydney, Australia; 11 Spine and Orthopedics, 293218 Chong Hua Hospital, Cebu, Philippines; 12 13143 Department of Orthopaedic Surgery and Spinal Surgery, The University of Tokyo Hospital, Tokyo, Japan; 13 Division of Neurosurgery, Department of Surgery, University Malaya Medical Centre, Petaling Jaya, Malaysia; 14 Combined Neurosurgical and Orthopedic Spine Program, Vancouver General Hospital, University of British Columbia, Vancouver, BC, Canada; 15 Department of Neurological Surgery, 6559 Thomas Jefferson University, Philadelphia, PA, USA.; 16 Division of Neurosurgery, Department of Surgery, 7938 University of Toronto, Toronto, ON, Canada; 17 Department of Orthopaedics, University of British Columbia, Vancouver, BC, Canada; 18 Department of Neurological Surgery, 8789 University of California Davis, Sacramento, CA, USA; 19 Department of Neurology, 37748 University Hospital Brno and Masaryk University, Brno, Czech Republic; 20 Division of Neurosurgery, Department of Clinical Neurosciences, 2152 University of Cambridge, Cambridge, UK

**Keywords:** cervical myelopathy, spinal cord compression, ossification of the posterior longitudinal ligament, spondylosis, disc herniation, cervical stenosis, spinal stenosis

## Abstract

**Study Design:**

Systematic review.

**Objectives:**

The pre-symptomatic state of Degenerative Cervical Myelopathy (DCM), wherein degenerative changes and spinal cord compression are seen without clinical findings, is poorly understood and inconsistently categorised. Clear identification may elucidate the temporality of DCM development. Therefore, a systematic assessment was undertaken of current terminology for pre-DCM states, with the objective of standardising definitions and informing an AO Spine expert position statement.

**Methods:**

Medline and Embase were searched for all studies on asymptomatic spinal compression or clinical findings preceding DCM, returning 3585 studies. After screening, 96 studies were included in the final analysis. The terminology used for pre-DCM states and their definitions were extracted, along with their frequencies or speciality/country of author in the literature.

**Results:**

Multiple terms were used to represent pre-DCM stages, including “*asymptomatic*” (86 studies), “*non-myelopathic*” (26 studies), “without myelopathy” (15 studies), “*pre-symptomatic*” (9 studies) and “*sub-clinical*” (7 studies). “*asymptomatic*” was associated with the greatest inconsistency. Some defined this as patients with radiological signs of spinal degeneration with/without spinal cord compression but no clinical signs of myelopathy, whereas others used the term synonymously with healthy controls. This inconsistency is particularly challenging in clinical studies in which DCM patients are compared to those with pre-DCM states and/or healthy volunteers.

**Conclusion:**

There is substantial inconsistency in the terms used to describe pre-DCM states. There is no clear relationship between the terms used and the country or speciality of the main author. Standardised definitions for these disease states should be agreed and used in future studies.

## Introduction

Degenerative Cervical Myelopathy (DCM) is a degenerative condition of symptomatic spinal cord compression. It is an umbrella term introduced by Nouri et al. that encompasses various degenerative spinal pathologies such as cervical spondylotic myelopathy (CSM), degenerative disc disease (DDD), ossification of the posterior longitudinal ligament (OPLL) and ossification of ligamentum flavum (OLF). These conditions are highly interrelated and often manifest concomitantly. DCM is a clinical diagnosis that relies on patient’s symptoms and clinical signs such as upper limb numbness/paraesthesia, loss of manual dexterity, gait abnormalities, hyperreflexia and Hoffman sign to be present alongside radiological evidence of spinal cord compression.^1-3^

Patients are assessed and stratified using the modified Japanese Orthopaedic Association scale (mJOA), which produces a score dependent on the extent of motor and sensory dysfunction in the upper and lower limbs as well as bladder sphincter dysfunction.^3,4^ The AO Spine clinical guidelines recommend surgical intervention for patients with moderate to severe or progressive DCM,^5^ based on a series of relatively recent papers that provide clear supporting evidence.^6^ For patients with mild DCM, there is a lack of high-quality research that limits the strength of recommendation, with either a trial of non-operative management or surgical intervention proposed.^5^ These guidelines do not recommend prophylactic surgical treatment of patients with spinal cord compression without signs or symptoms of myelopathy and radiculopathy.

The understanding of the pathogenesis of DCM and its progression from neurologically intact to a myelopathic condition is poorly understood. This may be because from a radiological perspective, various changes can represent pre-DCM pathologies: OPLL or OLF without cord compression and cord compression with or without cord signal change but without clinical myelopathic symptoms.^1,2^ From a clinical perspective, individuals may present with cervical radiculopathy or upper motor neurone signs such as Hoffman sign or hyperreflexia. The numerous clinical and radiographic findings have resulted in various terminologies that categorize the same population – individuals at risk of eventually developing DCM.

Multiple studies have been conducted on these patients and have demonstrated a significant prevalence of pre-DCM degenerative spinal pathology. Nakashima et al. recruited 1211 healthy neurologically intact volunteers consisting of 100 men and 100 women from each decade between 20 to 70 years. They found a prevalence of 5.3% for spinal cord compression (SCC) and 2.3% for increased signal intensity on MRI^7^. In a meta-analysis of MRI reports, Smith et al found the prevalence of spinal cord compression to be 24.2% in healthy volunteers. Additionally, they found the prevalence of DCM to be 2.3% within a subgroup of the healthy cohort.^8^ Martin et al. used objective spinal cord morphology metrics and found that spinal cord deformation (flatting, indentation, torsion, or circumferential compression) occurred in 50% of healthy subjects, increasing linearly with age.^9^

However, studies addressing pre-DCM pathology demonstrate inconsistency in descriptive terminology, including in the use of “*asymptomatic*”, “*pre-symptomatic*” and “*non-myelopathic*”. This is a result of a lack of standardized terminology for describing the preceding stages of the DCM entity. This gap impedes cross-comparisons between studies, and correct patient identification, ultimately hindering the assessment of risk factors and the temporality of DCM development. Clinically, this is relevant as prompt and early diagnosis of DCM is associated with better outcomes.^3,10^

The objective of this systematic review was therefore to synthesise the current terminology and definitions used in the literature for pre-DCM pathologies with the aim of forming the basis for an AO Spine expert position statement on standardised definitions for this clinical entity.

## Methods

### Study Design

A systematic review was conducted with reference to the Preferred Reporting Items for Systematic Reviews and Meta-Analyses (PRISMA) 2020 checklists (Online Appendix C).^11,12^ The protocol was registered on PROSPERO (ID: CRD42022307883).

### Eligibility Criteria

#### Inclusion Criteria

• Human study
• Adult (>18 years old)
• English language
• Full-text available
• Primary research paper, review or opinion article
• Discussion of pre-symptomatic or early stage of any degenerative cervical spinal pathology or condition within the DCM umbrella
• Published in the last 12 years (2012-2024)

#### Exclusion Criteria

• Animal studies
• Cadaveric studies
• Case reports
• Conference abstracts

### Information Sources

MEDLINE and Embase were searched using the Ovid Platform from inception to October 2024.

### Search Strategy

Scoping searches were performed to refine the review question. Final search strategies (Online Appendix A) were developed and piloted using an iterative process. The search included multiple possible terms used to describe pre-DCM, as well as subject headings to maximise search sensitivity. During the preliminary research stage, 10 studies were identified as significantly relevant to the study (Online Appendix B). These were used to test the search strategy sensitivity by ensuring all 10 studies were captured by the final search. Only studies published in the last 12 years were included to ensure focus on the most contemporaneous terminology.

### Selection Process

Search results were exported and deduplicated using EndNote (Version 20.3.0.17787, Clarivate, London, United Kingdom).^13^ Title and abstract screening was completed using Rayyan (Rayyan Systems Inc, Cambridge, MA, United States). All records were screened in duplicate by two blinded reviewers; a pilot of 100 records was screened by all reviewers to ensure concordance. Full-text screening was completed in duplicate.

### Data Collection

Manual data extraction from the 96 studies that passed screening was completed in duplicate in Microsoft Excel 2016 (Version 2311, Microsoft 365).

### Data Items

Data was independently extracted on the various terminology used and their definitions. Where clinical studies were included, further information was sought regarding their methodology. Specifically, this included the study design, recruitment process, inclusion and exclusion criteria, patient demographics (age, gender, ethnicity), mode of imaging and the overall conclusion.

### Risk of Bias Assessment

The quality and bias of included studies were assessed using appropriate Joanna Briggs Institute (JBI) critical appraisal tools depending on the type of literature: systematic review, cohort studies, cross-sectional studies and case-control studies. For review articles, the Scale for the Assessment of Narrative Review Articles (SANRA) was used, with which a score was given for each of its six criteria. To make the data comparable across different appraisal tools, an overall score was given (as a percentage) based on the specific criteria fulfilled by each study. Two authors assessed the quality and bias of these studies independently.

### Synthesis Methods

The frequency of different terminology in the literature was calculated using Microsoft Excel and the respective definitions for each of the terms were analysed. As the data was entirely qualitative, the scope for conducting extensive statistical analyses was limited.

## Results

**Figure 1.**
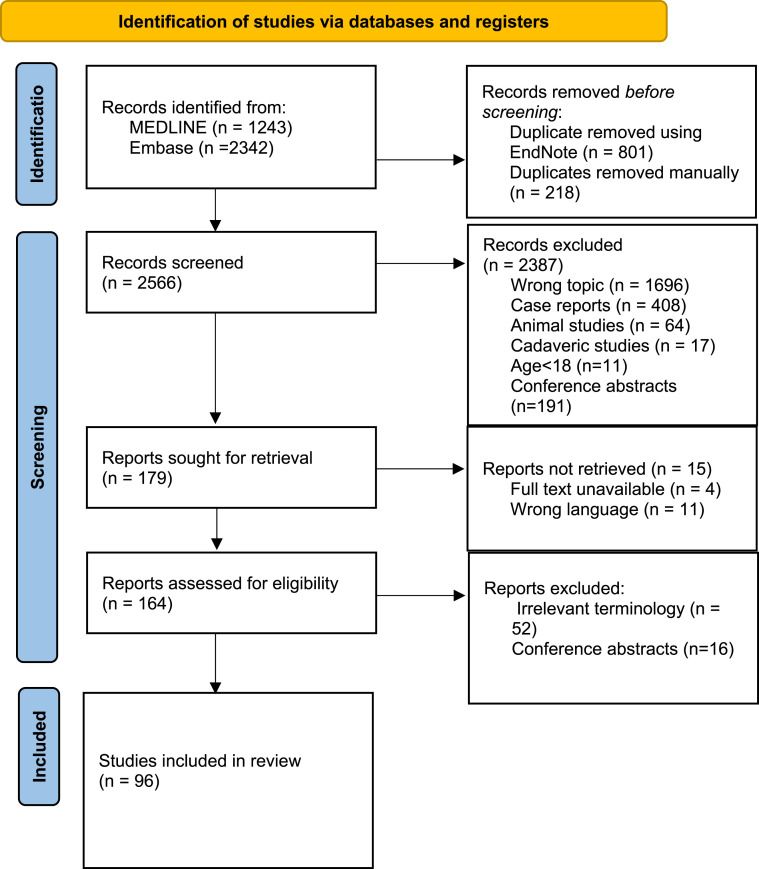
Preferred Reporting Items for Systematic Reviews and Meta-Analyses (PRISMA) flow diagram of study selection.

A total of 3585 records were identified by the search. Following removal of duplicates, 2566 records were screened against eligibility criteria. A total of 179 were sought for retrieval; 15 of these studies were not retrieved due to unavailability of full texts or because they were non-English language. During full text screening, 52 further studies were excluded as they did not focus on the early stages of DCM,^1,14-17^ and 16 further studies were excluded for being conference abstracts. A total of 96 studies were included in the final review (Figure 1; Online Appendix D).

The terms used in the literature to describe pre-DCM degenerative spinal pathology are presented in Table 1. The three most common terms were *asymptomatic*, *non-myelopathic* and *pre-symptomatic*. There was a discrepancy between the number of studies using a term and the frequency of use of terms across all studies, reflecting the same term being used in multiple contexts within individual studies and individual studies using different terms for the same concept.

**Table 1.** Summary of the Terms Used to Describe Pre-DCM States.

Term	Frequency of Use	Number of Studies
Asymptomatic	123	86
Non-myelopathic	30	26
Without myelopathy	15	15
Pre-symptomatic	9	9
Sub-clinical	7	7
Clinically silent	2	2
Minimal symptoms	2	2
No symptoms	2	2
Pre-clinical	1	1
Pre-myelopathic	1	1
Degenerative cervical spine changes without cord compression	1	1
Myelopathy-free	1	1
Without myelopathic signs and symptoms	1	1
OPLL patients with no or “slight” myelopathy	1	1
Lacking symptomatic myelopathy	1	1
Minimal OPLL	1	1
Subacute DCM	1	1

The *asymptomatic* terminology was applied in various contexts (Table 2). While *asymptomatic DCM* itself was only used twice,^18,19^ *asymptomatic* was used in the context of constituent conditions that fall under the umbrella of DCM, including cervical spondylotic myelopathy. Furthermore, in some studies *asymptomatic* was used to describe healthy volunteers and control groups.^7,20,21^ However, in other studies it was used to describe patients who had radiological evidence of degenerative spinal pathology with or without spinal cord compression but lacked neurological symptoms.^10,22-24^ *Asymptomatic* was often used interchangeably with other terms such as *non-myelopathic*^25,26^ or *pre-symptomatic*.^23^

**Table 2.** Further Analysis of the Use of the Term Asymptomatic.

Term	Frequency
Asymptomatic subjects/ individuals/adults	36
Asymptomatic cervical spinal cord compression	32
Asymptomatic cervical canal stenosis	15
Asymptomatic OPLL	12
Asymptomatic cervical spondylosis	7
Asymptomatic cervical spondylotic myelopathy	4
Other uses	4
Asymptomatic lesions / structural abnormalities	3
Asymptomatic DCM	2
Asymptomatic cervical disc degeneration	2
Asymptomatic degenerative cervical cord compression	2
Asymptomatic cervical spondylotic stenosis	1
Asymptomatic myelopathy	1

The second most frequently used term to describe pre-DCM states was *non-myelopathic*. There were fewer variations in the application of this term (Table 3), attributable to the extent to which symptoms were present. In some studies, patients had radiological evidence of spinal cord compression but no clinical symptoms or signs of myelopathy.^26-28^ In some studies, manifestations of radiculopathy were permitted for categorisation as *non-myelopathic*.^29^ In some cases, electrophysiological evidence was considered amongst other variables in the definition of *non-myelopathic*.^30^

**Table 3.** Breakdown of the Use of the Term Non-myelopathic.

Term	Frequency
Non-myelopathic patients	9
Non-myelopathic degenerative cervical cord compression (NMDCC)	7
Non-myelopathic with cord compression	5
Non-myelopathic OPLL	3
Non-myelopathic spinal cord compression	2
Non-myelopathic cervical cord compression	1
Non-myelopathic patients with cervical stenosis	1
Compression of a non-myelopathic cord	1
Non-myelopathic symptoms	1

**Figure 2.**
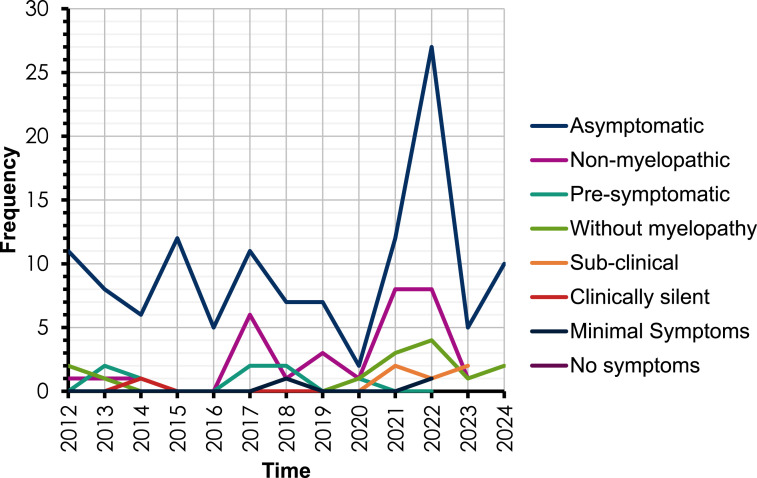
The frequency of different terms used to describe pre-DCM states from 2012-2024.

There were no obvious temporal trends in use of terminology during the period studied (Figure 2). The frequency of use of *asymptomatic* fluctuated from year to year and *non-myelopathic* became more prevalently used from 2016 onwards. The use of *asymptomatic* is the most common term used to describe pre-DCM states.

**Figure 3.**
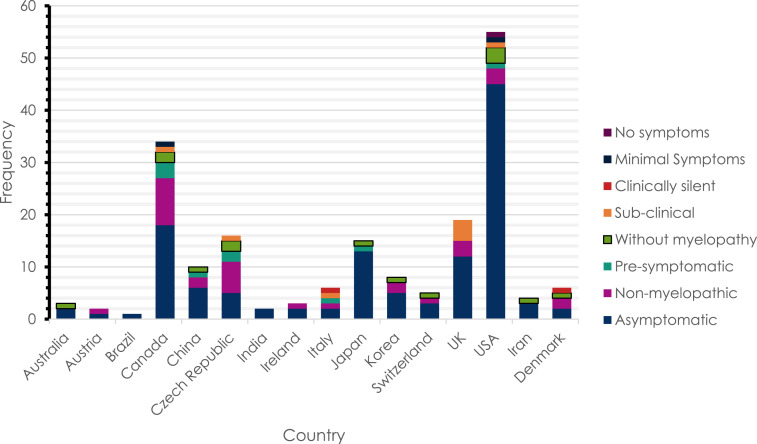
The absolute frequency of different terms used to describe pre-DCM states in different countries.

Assessment of pre-DCM terminology geographical variation showed that the United States and Canada produced the greatest volume of literature on the subject. The term *asymptomatic* appeared more prevalent in the literature emanating from the United States, whereas greater variation was present amongst Canadian studies (Figure 3).

**Figure 4.**
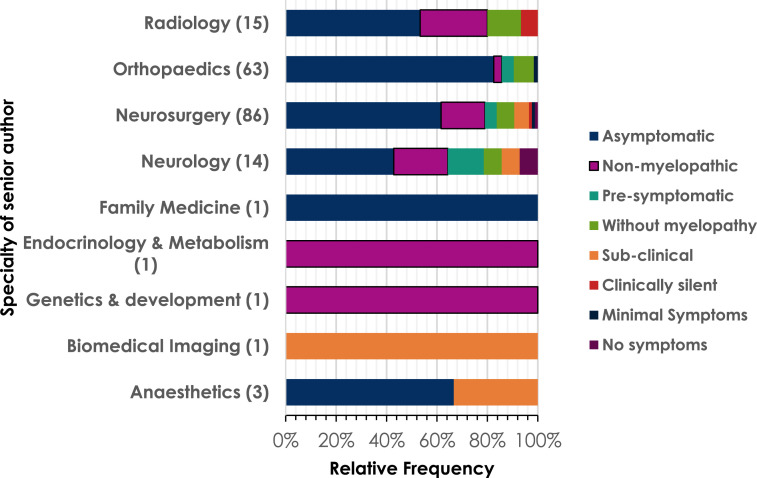
The relative frequency of different terms used to describe pre-DCM states by different specialities. Total numbers of papers from each specialty included in brackets after specialty name.

The included literature was found to be written by authors who were primarily from a neurosurgical or orthopaedic background. A broader range of terms were used by neurosurgeons than orthopaedic surgeons (Figure 4).

**Figure 5.**
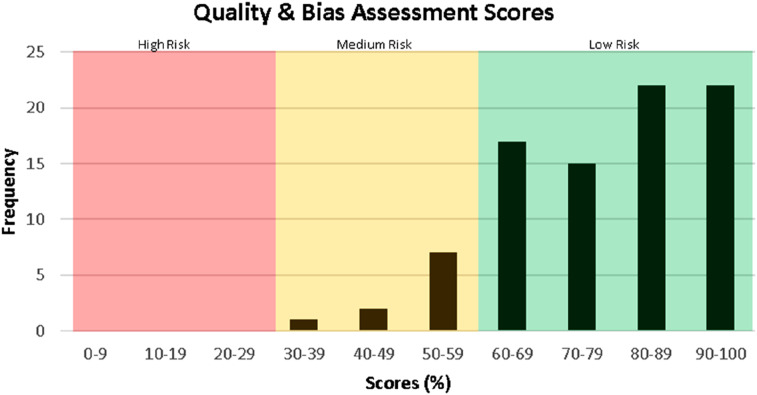
Frequency table of the quality and bias assessment scores for the studies included in data extraction.

Most studies were high quality with low bias (Figure 5). Studies in the medium risk category were still included in this study because regardless of their quality, the terminology used was still relevant.

## Discussion

Whilst the conditions encompassed by DCM have been the subject of considerable study, there is significantly less literature addressing the stages preceding DCM. Thus far, research on the terminology used to describe these stages has not been conducted. The discussion that does exist around the semantics of the field is brief, and is usually included at the end of papers, with clear definitions of terminology uncommon. The aim of this review was therefore to synthesise the definitions used and identify the inconsistencies in the literature.

The most used term to describe a pre-DCM state was *asymptomatic*, which has been primarily used in two ways. One use is with reference to healthy individuals without any prior known evidence of disease; many authors have used this term to describe the control group in their clinical studies. For example, Nakashima et al, 2016, described 1211 *asymptomatic* healthy volunteers without neurological symptoms, 5.3% of whom were found to have incidental spinal cord compression. Another study to exemplify this was Machino et al, 2019; a group of CSM patients were compared to a control group of *asymptomatic* patients to establish the clinical cut-off for two measures; the 10-second grip and release test and the step test.^31^ Similarly, Jaumard et al, 2014 used *asymptomatic* to describe a control group of 10 healthy volunteers when comparing the thickness and volume of the facet joint space of the cervical spine to eight symptomatic patients with cervical radiculopathy.^21^

The second use of *asymptomatic* in pre-DCM literature pertained to “individuals who have evidence of cord compression on imaging but do not have myelopathic symptoms”.^10^ This was supported by Wang et al, 2021, who defined asymptomatic spondylotic cervical spinal stenosis (ASCSS) as the “pre-symptomatic stage of chronic degenerative cervical spinal cord compression” with patients often presenting with “radiological evidence of compression but without evident symptoms”.^22^ Further endorsement for this definition was provided by Cao et al, 2017, in which 68 patients with mild or moderate spinal cord compression were subdivided into an asymptomatic group, that had no symptoms or signs of myelopathy, and a symptomatic group.^23^ Davies et al., 2023 describe two pre-DCM stages being conceptualised in the wider literature, with the onset of DCM effectively being the transition from degenerative spinal changes without cord compression, to asymptomatic/subclinical cord compression, to clinically symptomatic cervical cord compression.^32^ Nonetheless Davies et al acknowledge that this description is not necessarily simple or linear given compression is itself a poor predictor of conversion to DCM, as demonstrated with various clinical studies.^33-35^ Therefore, the use of asymptomatic is vague and unable to denote what degenerative/morphological changes that have occurred in the cervical spinal cord for those asymptomatic subjects.

Another term used with variability in the literature was *non-myelopathic*. Generally, it was defined as “patients with spinal canal narrowing or spinal cord compression who do not have clinical signs and symptoms of myelopathy (non-myelopathy spinal cord compression (NMSCC)).”.^26^ However, within this group, some had no neurological symptoms at all, whereas others had clinical manifestations of radiculopathy.^27^ This definition was employed by Du et al, 2019 who retrospectively reviewed 68 patients with OPLL who presented with radiculopathy.^27^ This group was divided into “myelopathy” and “non-myelopathy” subgroups based on clinical examination findings. Similarly, Kadanka et al, 2021 conducted a cross-sectional observation study comparing gait between DCM patients, non-myelopathic degenerative cervical cord compression (NMDCC) patients and healthy volunteers.^36^ The NMDCC group were patients with “MRI signs of cervical cord compression” who “may have exhibited one clinical myelopathic symptom” but “were free of clinical myelopathic signs and/or lacked the combination of one clinical symptom and one clinical sign of symptomatic myelopathy required for a diagnosis of DCM”. These studies of *non-myelopathic* groups, highlight the variability in patient presentations amongst those classified to be in a pre-DCM state.

While there were studies that used the terms *non-myelopathic* and *asymptomatic* interchangeably to describe a patient with no symptoms, Kadanka et al, 2017 discussed the difference between those terms when reflecting on the limitations of their previous study. They state that although their patients lacked any clear myelopathic symptoms and/or signs (ie, were “non-myelopathic”), they were not completely asymptomatic as they had radiculopathy and/or axial cervical pain.^37^ They suggested that “asymptomatic degenerative cervical cord compression” was reserved for completely asymptomatic cases, while “non-myelopathic degenerative cervical cord compression” was used specifically for patients who lack myelopathic symptoms but may or may not have radiculopathy or cervical pain. Complicating this matter is the possibility that a radicular pattern of symptoms occurs due to focal compression of the spinal cord at or near the root entry, which may be termed myelo-radiculopathy. Kadanka et al. (2024), however, referred to the presence of MRI findings of degenerative cervical cord compression without signs and symptoms of myelopathy as “asymptomatic or non-myelopathic degenerative cervical cord compression (ADCC, NMDCC)” without making the explicit distinction between the two, emphasising the importance in ensuring a consensus statement is made in distinguishing between the two terms.^45^ It should be noted, however, that Kadanka et al, 2024 did later make the distinction between “patients with eiter non-myelopathic symptoms (radiculopathy, cervical pain) or completely asymptomatic subjects (in whom cervical cord compression was found in volunteers during the epidemiological study or incidentally)”, however the inclusion of epidemiological studies or incidental findings add extra complexity to the definition that must be sorted to ensure the definition of asymptomatic DCM is clear.

Additional terms that have been used to describe patients in the stage preceding DCM are presented in Table 1. An example is the term *pre-symptomatic*, defined by Cao et al, 2017 as patients who “lack the typical symptoms and signs of upper motor neuron damage caused by spinal cord compression, apart from neck and shoulder pain, nerve root irritation and limited neck range of movement”.^23^ This definition was influenced by the work of Bednarik et al, 2008, who used other synonymous terms to *pre-symptomatic* such as *clinically silent* and *subclinical*.^38^ However, these terms are less commonly used throughout the literature.

It is possible that the inconsistency found between authors describing pre-DCM states stems from the uncertainty in the meaning of *myelopathy*. Despite it being well understood that DCM is a clinical diagnosis, *myelopathy* in isolation has variably been used to specifically describe radiological evidence of injury of the spinal cord due to compression, or in the more functional sense as the presence of symptoms and signs associated with spinal cord dysfunction.^9,39^ Whilst *myelopathy* is often used from a radiological perspective, most authors would describe myelopathy as a clinical diagnosis and spinal cord compression as a radiological diagnosis.^27^ This leads to obvious redundancies in the meanings of *symptomatic DCM* and *symptomatic myelopathy* which are frequently used in the literature. Furthermore, Farahbakhsh et al, 2023 present evidence that with spinal cord compression but no evident signs of myelopathy may still demonstrate electrophysiological markers of central conduction deficit and can then progress to myelopathy. This demonstrates existence of a pre-DCM state with evidence of a spinal cord “dysfunction”.^38,40,41^ Therefore, it is important that *myelopathy* is well-defined and used consistently because it influences the interpretations of other terms in the literature.

Moreover, there is variability in patients’ presentations in the pre-DCM state and this influences the terms used to describe these stages. Many clinical studies investigating conditions preceding DCM had different inclusion and exclusion criteria making it difficult to reproduce or compare their findings. For example, in a prospective cohort study of 115 patients with non-myelopathic cervical OPLL, 23 had an absence of symptoms, 44 had axial neck pain, 40 had radiculopathy and eight had tingling sensation of the fingers.^29^ In contrast, a retrospective cohort study by Shen et al, 2021, excluded patients from their non-myelopathic group if they had neurological deficits, symptoms of radiculopathy, cervical axial pain, previous spinal surgery, or a diagnosis of either spondylolisthesis or scoliosis.^42^ Furthermore, healthy subjects and individuals with DCM frequently report intermittent symptoms (eg, numbness), and most studies do not address how this finding is reported or categorized. Some studies also reported abnormalities in electrophysiological testing in asymptomatic individuals, thus adding to this diversity in pre-DCM conditions.^38,43^ Nouri et al. argue that both the variations in the diagnostic criteria and differences between ethnic populations may explain the heterogeneity in the prevalence rates of “non-myelopathic spinal cord compression”.^26^

## Future Directions

The RECODE-DCM Natural History Incubator is leading an AO Spine terminology consensus statement. These standardised definitions will allow better characterisation of the pathology preceding DCM, facilitate more efficient stratification of this large group of patients and promote optimal clinical management and research efficiency. A recent cross-sectional observational study, involving a web-based survey targeted towards surgeons and other health care professionals involved with DCM patients, investigated definitions of DCM and standard practice in DCM management.^44^ They found no consensus on the definitions and management of pre- or mild DCM and deficient follow-up assessments. Brannigan et al, 2024 discuss that inconsistent use of diagnostic terms can give rise to ambiguity when exploring diagnoses and interventions for patients. It is important to note that these results were found despite some authors having offered an approach to the management of patients in the pre-DCM stage, such as Fehlings et al, 2017. They suggest counselling these patients about the risks of progression, educating them about the signs and symptoms of myelopathy and ensuring clinical follow-up.^5^ For patients with additional symptoms of radiculopathy, they suggest informing them of the increased risk of myelopathy development and offering either surgical intervention, close regular follow-up or a trial of structured rehabilitation. The strength of these recommendations was weak due to being based on limited evidence, thus further highlighting the need for more research in this field with consistent terminology.

## Limitations

Limitations of this study include pre-DCM terminology not being clearly defined by many authors and so the meaning of the terms had to be inferred by the context, the study design and the inclusion and exclusion criteria. This was negated somewhat in our methodology by having two independent authors reviewing the literature. Limitations also arose due to the exclusion of non-English literature. This resulted in a lack of data of equivalent terms in other countries, which may have affected the results shown in Figure 3. Finally, this research is limited in that we can only form conclusions about the variable usage of terms describing pre-DCM states in the academic literature and do not specifically capture descriptors employed in clinical practice.

## Conclusion

There are many terms in the literature used to describe conditions preceding DCM, with the most common being *asymptomatic*, *non-myelopathic, without myelopathy* and *pre-symptomatic*. These are often used interchangeably, however, there is also heterogeneity within the definitions of each of these terms. There is a large amount of variability in the presentation of patients before their diagnosis of DCM, giving rise to the different inclusion and exclusion criteria of clinical studies. The aim of this project was to inform an AO Spine consensus statement on standardised terminology for pre-DCM states. The aim is that this will increase reproducibility between studies and aid the clinical management of these patients.

## Supplemental Material

Supplemental Material - A Systematic Review of Current Terminology for Conditions Preceding Degenerative Cervical Myelopathy: Evidence Synthesis to Inform an AO Spine Expert Opinion Statement Supplemental Material for A Systematic Review of Current Terminology for Conditions Preceding Degenerative Cervical Myelopathy: Evidence Synthesis to Inform an AO Spine Expert Opinion Statement by Vinisha Agrawal, Mohammed Farhan Ali, Froher Yasin, Daniyal Ashraf, Jamie FM Brannigan, Ratko Yurac, MD, Vishal Kumar, Rory Murphy, Enrico Tessitore, Granit Molliqaj, Joost Dejaegher, Juan José Zamorano, Guy Wynne-Jones, Manjul Tripathi, David B Anderson, Jose Joefrey F Arbatin, So Kato, Ronie Romelean Jayapalan, Nicolas Dea, James S. Harrop, Jefferson Wilson, Brian K. Kwon, Allan R. Martin, Josef Bednarik, Mark R Kotter, Benjamin M Davies, Oliver D Mowforth, and Aria Nouri in Global Spine Journal.

## ORCID iDs

Mohammed Farhan Ali https://orcid.org/0009-0005-4776-7596

Jamie FM Brannigan https://orcid.org/0000-0003-1697-403X

Ratko Yurac https://orcid.org/0000-0003-3603-6294

Joost Dejaegher https://orcid.org/0000-0002-0050-5485

Juan José Zamorano https://orcid.org/0000-0002-7284-0784

Jose Joefrey F Arbatin https://orcid.org/0000-0002-1577-7977

Jefferson Wilson https://orcid.org/0000-0001-5965-0305

Josef Bednarik https://orcid.org/0000-0001-7420-2383

Benjamin M Davies https://orcid.org/0000-0003-0591-5069

Oliver D Mowforth https://orcid.org/0000-0001-6788-745X

Aria Nouri https://orcid.org/0000-0002-4965-3059
