# Supplementary material for: A Systematic Review of Current Terminology for Conditions Preceding Degenerative Cervical Myelopathy: Evidence Synthesis to Inform an AO Spine Expert Opinion Statement
Source: Global Spine J. 2025 Apr 30;15(7):3506–16. doi: 10.1177/21925682251339480 (PMC12043630; doi:10.1177/21925682251339480)
Supplement: Supplemental Material - A Systematic Review of Current Terminology for Conditions Preceding Degenerative Cervical Myelopathy: Evidence Synthesis to Inform an AO Spine Expert Opinion Statement [file sj-pdf-1-gsj-10.1177_21925682251339480.pdf]

Appendices

Appendix A: Search Strategy

1. (Asymptomatic OR Non-symptomatic OR Pre-symptomatic OR Non-myelopathic OR Nonmyelopathic OR Symptomless OR “natural history”).ti,ab.
2. (“Degenerative cervical myelopathy” OR “Cervical spondylosis” OR “Cervical spondylotic myelopathy” OR “Cervical myelopathy” OR “Spinal Cord Disease” OR “chronic spinal cord injury”).ti,ab. OR Spinal cord diseases/ OR spinal cord compression/
3. (Spine OR “Cervical cord” OR “Cervical spine” OR “Cervical spinal stenosis” OR “Spinal cord” OR Cervical).ti,ab. OR Cervical vertebrae/ OR spinal stenosis/
4. (Compression OR Spondylosis OR Spondylotic OR Impingement OR Stenosis OR “Diameter change”).ti,ab. OR spondylosis/
5. (Disc AND (Degeneration OR Herniation OR Protusion OR Annular tear OR Ossification OR Degenerative disease)).ti,ab. OR intervertebral disc degeneration/
6. (“Ossification of the posterior longitudinal ligament” OR “Ligament ossification” OR “Posterior longitudinal ligament” OR “Ligamentum flavum” OR “Ligament hypertrophy” OR “Ligament calcification” OR OPLL).ti,ab. OR “Ossification of the posterior longitudinal ligament”/
7. 3 AND (4 OR 5 OR 6)
8. 1 AND (2 OR 7)

Appendix B: List of studies to test the sensitivity of the search strategy.

Ten studies were identified during the preliminary research stage, as significantly relevant to the study. These studies were used to test the search strategy sensitivity by ensuring all 10 studies were picked up in the final search.

A. Smith SS, Stewart ME, Davies BM, Kotter MRN. The Prevalence of Asymptomatic and Symptomatic Spinal Cord Compression on Magnetic Resonance Imaging: A Systematic Review and Meta-analysis. Global Spine J. 2021 May
B. Tetreault L, Goldstein CL, Arnold P, Harrop J, Hilibrand A, Nouri A, Fehlings MG. Degenerative Cervical Myelopathy: A Spectrum of Related Disorders Affecting the Aging Spine. Neurosurgery. 2015 Oct
C. Nakashima H, Yukawa Y, Suda K, Yamagata M, Ueta T, Kato F. Narrow cervical canal in 1211 asymptomatic healthy subjects: the relationship with spinal cord compression on MRI. Eur Spine J. 2016 Jul
D. Badhiwala JH, Wilson JR. The Natural History of Degenerative Cervical Myelopathy. Neurosurg Clin N Am. 2018 Jan
E. Kato F, Yukawa Y, Suda K, Yamagata M, Ueta T. Normal morphology, age-related changes and abnormal findings of the cervical spine. Part II: Magnetic resonance imaging of over 1,200 asymptomatic subjects. Eur Spine J. 2012 Aug
F. Wilson JR, Barry S, Fischer DJ, Skelly AC, Arnold PM, Riew KD, Shaffrey CI, Traynelis VC, Fehlings MG. Frequency, timing, and predictors of neurological dysfunction in the nonmyelopathic patient with cervical spinal cord compression, canal stenosis, and/or ossification of the posterior longitudinal ligament. Spine (Phila Pa 1976). 2013 Oct 15.
G. Karadimas SK, Erwin WM, Ely CG, Dettori JR, Fehlings MG. Pathophysiology and natural history of cervical spondylotic myelopathy. Spine (Phila Pa 1976). 2013 Oct
H. Boody BS, Schroeder GD, Segar AH, Kepler CK. Should Asymptomatic Patients With Cervical Spinal Cord Compression and Spinal Cord Signal Change Undergo Surgical Intervention? Clin Spine Surg. 2019 Apr;32(3):87–90.
I. Cao JM, Zhang JT, Yang DL, Yang YP, Xia HH, Yang L. Imaging Factors that Distinguish Between Patients with Asymptomatic and Symptomatic Cervical Spondylotic Myelopathy with Mild to Moderate Cervical Spinal Cord Compression. Med Sci Monit. 2017 Oct 13;23:4901–8.
J. Nouri A, Tessitore E, Molliqaj G, Meling T, Schaller K, Nakashima H, et al. Degenerative Cervical Myelopathy: Development and Natural History [AO Spine RECODE-DCM Research Priority Number 2]. Global Spine J. 2022 Feb;12(1_suppl):39S-54S.

Appendix C: PRISMA 2020 Checklist

Section and Topic	Item #	Checklist item
**TITLE**		
Title	1	Identify the report as a systematic review.
**ABSTRACT**		
Abstract	2	See the PRISMA 2020 for Abstracts checklist.
**INTRODUCTION**		
Rationale	3	Describe the rationale for the review in the context of existing knowledge.
Objectives	4	Provide an explicit statement of the objective(s) or question(s) the review addresses.
**METHODS**		
Eligibility criteria	5	Specify the inclusion and exclusion criteria for the review and how studies were grouped for the syntheses.
Information sources	6	Specify all databases, registers, websites, organisations, reference lists and other sources searched or consulted to identify studies. Specify the date when each source was last searched or consulted.
Search strategy	7	Present the full search strategies for all databases, registers and websites, including any filters and limits used.
Selection process	8	Specify the methods used to decide whether a study met the inclusion criteria of the review, including how many reviewers screened each record and each report retrieved, whether they worked independently, and if applicable, details of automation tools used in the process.
Data collection process	9	Specify the methods used to collect data from reports, including how many reviewers collected data from each report, whether they worked independently, any processes for obtaining or confirming data from study investigators, and if applicable, details of automation tools used in the process.
Data items	10a	List and define all outcomes for which data were sought. Specify whether all results that were compatible with each outcome domain in each study were sought (e.g. for all measures, time points, analyses), and if not, the methods used to decide which results to collect.
	10b	List and define all other variables for which data were sought (e.g. participant and intervention characteristics, funding sources). Describe any assumptions made about any missing or unclear information.
Study risk of bias assessment	11	Specify the methods used to assess risk of bias in the included studies, including details of the tool(s) used, how many reviewers assessed each study and whether they worked independently, and if applicable, details of automation tools used in the process.
Effect measures	12	Specify for each outcome the effect measure(s) (e.g. risk ratio, mean difference) used in the synthesis or presentation of results.
Synthesis methods	13a	Describe the processes used to decide which studies were eligible for each synthesis (e.g. tabulating the study intervention characteristics and comparing against the planned groups for each synthesis (item #5)).
	13b	Describe any methods required to prepare the data for presentation or synthesis, such as handling of missing summary statistics, or data conversions.
	13c	Describe any methods used to tabulate or visually display results of individual studies and syntheses.
	13d	Describe any methods used to synthesize results and provide a rationale for the choice(s). If meta-analysis was performed, describe the model(s), method(s) to identify the presence and extent of statistical heterogeneity, and software package(s) used.
	13e	Describe any methods used to explore possible causes of heterogeneity among study results (e.g. subgroup analysis, meta-regression).
	13f	Describe any sensitivity analyses conducted to assess robustness of the synthesized results.
Reporting bias assessment	14	Describe any methods used to assess risk of bias due to missing results in a synthesis (arising from reporting biases).
Certainty	15	Describe any methods used to assess certainty (or confidence) in the body of evidence for an outcome.

Section and Topic	Item #	Checklist item
assessment		
**RESULTS**		
Study selection	16a	Describe the results of the search and selection process, from the number of records identified in the search to the number of studies included in the review, ideally using a flow diagram.
	16b	Cite studies that might appear to meet the inclusion criteria, but which were excluded, and explain why they were excluded.
Study characteristics	17	Cite each included study and present its characteristics.
Risk of bias in studies	18	Present assessments of risk of bias for each included study.
Results of individual studies	19	For all outcomes, present, for each study: (a) summary statistics for each group (where appropriate) and (b) an effect estimate and its precision (e.g. confidence/credible interval), ideally using structured tables or plots.
Results of syntheses	20a	For each synthesis, briefly summarise the characteristics and risk of bias among contributing studies.
	20b	Present results of all statistical syntheses conducted. If meta-analysis was done, present for each the summary estimate and its precision (e.g. confidence/credible interval) and measures of statistical heterogeneity. If comparing groups, describe the direction of the effect.
	20c	Present results of all investigations of possible causes of heterogeneity among study results.
	20d	Present results of all sensitivity analyses conducted to assess the robustness of the synthesized results.
Reporting biases	21	Present assessments of risk of bias due to missing results (arising from reporting biases) for each synthesis assessed.
Certainty of evidence	22	Present assessments of certainty (or confidence) in the body of evidence for each outcome assessed.
**DISCUSSION**		
Discussion	23a	Provide a general interpretation of the results in the context of other evidence.
	23b	Discuss any limitations of the evidence included in the review.
	23c	Discuss any limitations of the review processes used.
	23d	Discuss implications of the results for practice, policy, and future research.
**OTHER INFORMATION**		
Registration and protocol	24a	Provide registration information for the review, including register name and registration number, or state that the review was not registered.
	24b	Indicate where the review protocol can be accessed, or state that a protocol was not prepared.
	24c	Describe and explain any amendments to information provided at registration or in the protocol.
Support	25	Describe sources of financial or non-financial support for the review, and the role of the funders or sponsors in the review.
Competing interests	26	Declare any competing interests of review authors.
Availability of data, code and other materials	27	Report which of the following are publicly available and where they can be found: template data collection forms; data extracted from included studies; data used for all analyses; analytic code; any other materials used in the review.

From: Page MJ, McKenzie JE, Bossuyt PM, Boutron I, Hoffmann TC, Mulrow CD, et al. The PRISMA 2020 statement: an updated guideline for reporting systematic reviews. BMJ 2021;372:n71. doi: 10.1136/bmj.n

Appendix D. List of studies included in the final review

1. Abiola R, Rubery P, Mesfin A. Ossification of the Posterior Longitudinal Ligament: Etiology, Diagnosis, and Outcomes of Nonoperative and Operative Management. Global Spine J. 2016 Mar;6(2):195-204. doi: 10.1055/s-0035-1556580. Epub 2015 Jun 30. PMID: 26933622; PMCID: PMC4771496.
2. Aljuboori Z, Boakye M. The Natural History of Cervical Spondylotic Myelopathy and Ossification of the Posterior Longitudinal Ligament: A Review Article. Cureus. 2019 Jul 3;11(7):e5074. doi: 10.7759/cureus.5074. PMID: 31516784; PMCID: PMC6721920.
3. Arkaprabha Banerjee, Oliver D. Mowforth, Aria Nouri, Alexandru Budu, Virginia Newcombe, Mark R.N. Kotter, Benjamin M. Davies. The Prevalence of Degenerative Cervical Myelopathy-Related Pathologies on Magnetic Resonance Imaging in Healthy/Asymptomatic Individuals: A Meta-Analysis of Published Studies and Comparison to a Symptomatic Cohort, Journal of Clinical Neuroscience, Volume 99, 2022, Pages 53-61.
4. Bakhsh W, Saleh A, Yokogawa N, Gruber J, Rubery PT, Mesfin A. Cervical Ossification of the Posterior Longitudinal Ligament: A Computed Tomography-Based Epidemiological Study of 2917 Patients. Global Spine J. 2019 Dec;9(8):820-825. doi: 10.1177/2192568219833658. Epub 2019 Mar 12. Erratum in: Global Spine J. 2021 Jul;11(6):1011. PMID: 31819847; PMCID: PMC6882099.
5. Barkoh K, Ohiorhenuan IE, Lee L, Lucas J, Arakelyan A, Ornelas C, Buser Z, Hsieh P, Acosta F, Liu J, Wang JC, Hah R. The DOWN Questionnaire: A Novel Screening Tool for Cervical Spondylotic Myelopathy. Global Spine J. 2019 Sep;9(6):607-612. doi: 10.1177/2192568218815863. Epub 2018 Dec 4. PMID: 31448193; PMCID: PMC6693067.
6. Cao JM, Zhang JT, Yang DL, Yang YP, Xia HH, Yang L. Imaging Factors that Distinguish Between Patients with Asymptomatic and Symptomatic Cervical Spondylotic Myelopathy with Mild to Moderate Cervical Spinal Cord Compression. Med Sci Monit. 2017 Oct 13;23:4901-4908. doi: 10.12659/msm.906937. PMID: 29028790; PMCID: PMC5652139.
7. Kato F, Yukawa Y, Suda K, Yamagata M, Ueta T. Normal morphology, age-related changes and abnormal findings of the cervical spine. Part II: Magnetic resonance imaging of over 1,200 asymptomatic subjects. Eur Spine J. 2012 Aug;21(8):1499-507. doi: 10.1007/s00586-012-2176-4. PMID: 22302162; PMCID: PMC3535246.
8. Nouri A, Tessitore E, Molliqaj G, Meling T, Schaller K, Nakashima H, Yukawa Y, Bednarik J, Martin AR, Vajkoczy P, Cheng JS, Kwon BK, Kurpad SN, Fehlings MG, Harrop JS, Aarabi B, Rahimi-Movaghar V, Guest JD, Davies BM, Kotter MRN, Wilson JR. Degenerative Cervical Myelopathy: Development and Natural History [AO Spine RECODE-DCM Research Priority Number 2]. Global Spine J. 2022 Feb;12(1_suppl):39S-54S. doi: 10.1177/21925682211036071. PMID: 35174726; PMCID: PMC8859703.
9. Smith SS, Stewart ME, Davies BM, Kotter MRN. The Prevalence of Asymptomatic and Symptomatic Spinal Cord Compression on Magnetic Resonance Imaging: A Systematic Review and Meta-analysis. Global Spine J. 2021 May;11(4):597-607. doi: 10.1177/2192568220934496. Epub 2020 Jun 24. PMID: 32677521; PMCID: PMC8119927.

10. Tetreault L, Goldstein CL, Arnold P, Harrop J, Hilibrand A, Nouri A, Fehlings MG. Degenerative Cervical Myelopathy: A Spectrum of Related Disorders Affecting the Aging Spine. Neurosurgery. 2015 Oct;77 Suppl 4:S51-67. doi: 10.1227/NEU.0000000000000951. PMID: 26378358.
11. Butler JS, Oner FC, Poynton AR, O'Byrne JM. Degenerative cervical spondylosis: natural history, pathogenesis, and current management strategies. Adv Orthop. 2012;2012:916987. doi: 10.1155/2012/916987. Epub 2012 Aug 27. PMID: 22966465; PMCID: PMC3433115.
12. Cuellar J, Passias P. Cervical Spondylotic Myelopathy A Review of Clinical Diagnosis and Treatment. Bull Hosp Jt Dis (2013). 2017 Jan;75(1):21-29. PMID: 28214458.
13. de Oliveira Vilaça C, Orsini M, Leite MA, de Freitas MR, Davidovich E, Fiorelli R, Fiorelli S, Fiorelli C, Oliveira AB, Pessoa BL. Cervical Spondylotic Myelopathy: What the Neurologist Should Know. Neurol Int. 2016 Nov 23;8(4):6330. doi: 10.4081/ni.2016.6330. PMID: 27994827; PMCID: PMC5136752.
14. Fehlings MG, Tetreault LA, Riew KD, Middleton JW, Aarabi B, Arnold PM, Brodke DS, Burns AS, Carette S, Chen R, Chiba K, Dettori JR, Furlan JC, Harrop JS, Holly LT, Kalsi-Ryan S, Kotter M, Kwon BK, Martin AR, Milligan J, Nakashima H, Nagoshi N, Rhee J, Singh A, Skelly AC, Sodhi S, Wilson JR, Yee A, Wang JC. A Clinical Practice Guideline for the Management of Patients With Degenerative Cervical Myelopathy: Recommendations for Patients With Mild, Moderate, and Severe Disease and Nonmyelopathic Patients With Evidence of Cord Compression. Global Spine J. 2017 Sep;7(3 Suppl):70S-83S. doi: 10.1177/2192568217701914. Epub 2017 Sep 5. PMID: 29164035; PMCID: PMC5684840.
15. Fontanella MM, Fazio M, Francione A, Bacigaluppi S, Griva F, Visocchi M, Panciani PP, Bergomi R, Spena G. Pre-symptomatic cervical myelopathy: should we operate or should we observe? What is the chance of spinal cord injury from an accident? J Neurosurg Sci. 2014 Jun;58(2 Suppl 1):15-22. PMID: 25371943.
16. Hejrati N, Moghaddamjou A, Marathe N, Fehlings MG. Degenerative Cervical Myelopathy: Towards a Personalized Approach. Can J Neurol Sci. 2022 Nov;49(6):729-740. doi: 10.1017/cjn.2021.214. Epub 2021 Oct 25. PMID: 34689848.
17. Bhattacharyya S. Spondylotic and Other Structural Myelopathies. Continuum (Minneap Minn). 2021 Feb 1;27(1):163-184. doi: 10.1212/CON.0000000000000975. PMID: 33522741.
18. Chen C, Huang M, Han Z, Shao L, Xie Y, Wu J, Zhang Y, Xin H, Ren A, Guo Y, Wang D, He Q, Ruan D. Quantitative T2 magnetic resonance imaging compared to morphological grading of the early cervical intervertebral disc degeneration: an evaluation approach in asymptomatic young adults. PLoS One. 2014 Feb 3;9(2):e87856. doi: 10.1371/journal.pone.0087856. PMID: 24498384; PMCID: PMC3912130.
19. Choi BW. Clinical and Radiological Characteristics of Ossification of the Posterior Longitudinal Ligament of the Cervical Spine in Patients without Myelopathy: Results of a 1-year Pilot Study. Turk Neurosurg. 2017;27(3):414-419. doi: 10.5137/1019-5149.JTN.16196-15.1. PMID: 27593790.
20. Du YQ, Duan WR, Chen Z, Wu H, Jian FZ. Predictors for Development of Symptomatic Myelopathy in Patients with Radiculopathy Caused by Cervical Ossification of Posterior Longitudinal Ligament.

World Neurosurg. 2019 Apr;124:e710-e714. doi: 10.1016/j.wneu.2018.12.199. Epub 2019 Jan 17. PMID: 30660887.

21. Ellingson BM, Salamon N, Woodworth DC, Yokota H, Holly LT. Reproducibility, temporal stability, and functional correlation of diffusion MR measurements within the spinal cord in patients with asymptomatic cervical stenosis or cervical myelopathy. J Neurosurg Spine. 2018 May;28(5):472-480. doi: 10.3171/2017.7.SPINE176. Epub 2018 Feb 9. PMID: 29424671; PMCID: PMC5930078.
22. Kadanka Z Jr, Kadanka Z Sr, Skutil T, Vlckova E, Bednarik J. Walk and Run Test in Patients with Degenerative Compression of the Cervical Spinal Cord. J Clin Med. 2021 Mar 1;10(5):927. doi: 10.3390/jcm10050927. PMID: 33804299; PMCID: PMC7957594.
23. Kim SJ, Lee TH, Yi S. Prevalence of disc degeneration in asymptomatic korean subjects. Part 3 : cervical and lumbar relationship. J Korean Neurosurg Soc. 2013 Mar;53(3):167-73. doi: 10.3340/jkns.2013.53.3.167. Epub 2013 Mar 31. PMID: 23634267; PMCID: PMC3638270.
24. Labounek R, Valošek J, Horák T, Svátková A, Bednařík P, Vojtíšek L, Horáková M, Nestrašil I, Lenglet C, Cohen-Adad J, Bednařík J, Hluštík P. HARDI-ZOOMit protocol improves specificity to microstructural changes in presymptomatic myelopathy. Sci Rep. 2020 Oct 16;10(1):17529. doi: 10.1038/s41598-020-70297-3. PMID: 33067520; PMCID: PMC7567840.
25. Sasaki E, Ono A, Yokoyama T, Wada K, Tanaka T, Kumagai G, Iwasaki H, Takahashi I, Umeda T, Nakaji S, Ishibashi Y. Prevalence and symptom of ossification of posterior longitudinal ligaments in the Japanese general population. J Orthop Sci. 2014 May;19(3):405-11. doi: 10.1007/s00776-014-0552-0. Epub 2014 Mar 5. PMID: 24595647.
26. Park MS, Moon SH, Kim TH, Oh JK, Lyu HD, Lee JH, Riew KD. Asymptomatic Stenosis in the Cervical and Thoracic Spines of Patients with Symptomatic Lumbar Stenosis. Global Spine J. 2015 Oct;5(5):366-71. doi: 10.1055/s-0035-1549031. Epub 2015 Mar 27. PMID: 26430589; PMCID: PMC4577327.
27. Shen J, McGraw M, Truong VT, Al-Shakfa F, Boubez G, Shedid D, Yuh SJ, Wang Z. C2-C3 vertebral disc angle: An analysis of patients with and without cervical spondylotic myelopathy. Neurochirurgie. 2021 Jul;67(4):346-349. doi: 10.1016/j.neuchi.2021.02.013. Epub 2021 Mar 20. PMID: 33757775.
28. Matsumoto M, Okada E, Toyama Y, Fujiwara H, Momoshima S, Takahata T. Tandem age-related lumbar and cervical intervertebral disc changes in asymptomatic subjects. Eur Spine J. 2013 Apr;22(4):708-13. doi: 10.1007/s00586-012-2500-z. Epub 2012 Sep 19. PMID: 22990606; PMCID: PMC3631032.
29. Nakashima H, Yukawa Y, Suda K, Yamagata M, Ueta T, Kato F. Abnormal findings on magnetic resonance images of the cervical spines in 1211 asymptomatic subjects. Spine (Phila Pa 1976). 2015 Mar 15;40(6):392-8. doi: 10.1097/BRS.0000000000000775. PMID: 25584950.
30. Keřkovský M, Bednařík J, Jurová B, Dušek L, Kadaňka Z, Kadaňka Z Jr, Němec M, Kovaľová I, Šprláková-Puková A, Mechl M. Spinal Cord MR Diffusion Properties in Patients with Degenerative Cervical Cord Compression. J Neuroimaging. 2017 Jan;27(1):149-157. doi: 10.1111/jon.12372. Epub 2016 Jun 16. PMID: 27307399.

31. Kanna RM, Kamal Y, Mahesh A, Venugopal P, Shetty AP, Rajasekaran S. The impact of routine whole spine MRI screening in the evaluation of spinal degenerative diseases. Eur Spine J. 2017 Aug;26(8):1993-1998. doi: 10.1007/s00586-017-4944-7. Epub 2017 Jan 21. PMID: 28110361.
32. Brinjikji W, Luetmer PH, Comstock B, Bresnahan BW, Chen LE, Deyo RA, Halabi S, Turner JA, Avins AL, James K, Wald JT, Kallmes DF, Jarvik JG. Systematic literature review of imaging features of spinal degeneration in asymptomatic populations. AJNR Am J Neuroradiol. 2015 Apr;36(4):811-6. doi: 10.3174/ajnr.A4173. Epub 2014 Nov 27. PMID: 25430861; PMCID: PMC4464797.
33. Iyer A, Azad TD, Tharin S. Cervical Spondylotic Myelopathy. Clin Spine Surg. 2016 Dec;29(10):408-414. doi: 10.1097/BSD.0000000000000397. PMID: 27352369.
34. Hejrati N, Moghaddamjou A, Marathe N, Fehlings MG. Degenerative Cervical Myelopathy: Towards a Personalized Approach. Can J Neurol Sci. 2022 Nov;49(6):729-740. doi: 10.1017/cjn.2021.214. Epub 2021 Oct 25. PMID: 34689848.
35. Milligan J, Ryan K, Fehlings M, Bauman C. Degenerative cervical myelopathy: Diagnosis and management in primary care. Can Fam Physician. 2019 Sep;65(9):619-624. PMID: 31515310; PMCID: PMC6741789.
36. Singh A, Tetreault L, Fehlings MG, Fischer DJ, Skelly AC. Risk factors for development of cervical spondylotic myelopathy: results of a systematic review. Evid Based Spine Care J. 2012 Aug;3(3):35-42. doi: 10.1055/s-0032-1327808. PMID: 23526904; PMCID: PMC3592758.
37. Matsunaga S, Komiya S, Toyama Y. Risk factors for development of myelopathy in patients with cervical spondylotic cord compression. Eur Spine J. 2015 Apr;24 Suppl 2:142-9. doi: 10.1007/s00586-013-2839-9. Epub 2013 May 23. PMID: 23700231.
38. Lebl DR, Bono CM. Update on the Diagnosis and Management of Cervical Spondylotic Myelopathy. J Am Acad Orthop Surg. 2015 Nov;23(11):648-60. doi: 10.5435/JAAOS-D-14-00250. PMID: 26498584.
39. Lannon M, Kachur E. Degenerative Cervical Myelopathy: Clinical Presentation, Assessment, and Natural History. J Clin Med. 2021 Aug 17;10(16):3626. doi: 10.3390/jcm10163626. PMID: 34441921; PMCID: PMC8396963.
40. Sheikh Taha AM, Shue J, Lebl D, Girardi F. Considerations for prophylactic surgery in asymptomatic severe cervical stenosis: review article. HSS J. 2015 Feb;11(1):31-5. doi: 10.1007/s11420-014-9426-4. Epub 2015 Jan 27. PMID: 25737666; PMCID: PMC4342391.
41. Tetreault LA, Karadimas S, Wilson JR, Arnold PM, Kurpad S, Dettori JR, Fehlings MG. The Natural History of Degenerative Cervical Myelopathy and the Rate of Hospitalization Following Spinal Cord Injury: An Updated Systematic Review. Global Spine J. 2017 Sep;7(3 Suppl):28S-34S. doi: 10.1177/2192568217700396. Epub 2017 Sep 5. PMID: 29164030; PMCID: PMC5684834.
42. Tu J, Vargas Castillo J, Das A, Diwan AD. Degenerative Cervical Myelopathy: Insights into Its Pathobiology and Molecular Mechanisms. J Clin Med. 2021 Mar 15;10(6):1214. doi: 10.3390/jcm10061214. PMID: 33804008; PMCID: PMC8001572.
43. Wilson JR, Tetreault LA, Kim J, Shamji MF, Harrop JS, Mroz T, Cho S, Fehlings MG. State of the Art in Degenerative Cervical Myelopathy: An Update on Current Clinical Evidence. Neurosurgery. 2017 Mar 1;80(3S):S33-S45. doi: 10.1093/neuros/nyw083. PMID: 28350949.

44. Witiw CD, Mathieu F, Nouri A, Fehlings MG. Clinico-Radiographic Discordance: An Evidence-Based Commentary on the Management of Degenerative Cervical Spinal Cord Compression in the Absence of Symptoms or With Only Mild Symptoms of Myelopathy. Global Spine J. 2018 Aug;8(5):527-534. doi: 10.1177/2192568217745519. Epub 2017 Dec 18. PMID: 30258760; PMCID: PMC6149046.
45. Yonenobu K. Is surgery indicated for asymptomatic or mildly myelopathic patients with significant ossification of the posterior longitudinal ligament? Spine (Phila Pa 1976). 2012 Mar 1;37(5):E315-7. doi: 10.1097/BRS.0b013e318239ccbd. PMID: 22020586.
46. Yarbrough CK, Murphy RK, Ray WZ, Stewart TJ. The natural history and clinical presentation of cervical spondylotic myelopathy. Adv Orthop. 2012;2012:480643. doi: 10.1155/2012/480643. Epub 2011 Dec 22. PMID: 22235378; PMCID: PMC3253434.
47. Wang Z, Rong Y, Tang P, Ye W, Ji C, Wang J, Ge X, Liu W, Li Q, Cai W. Prevalence and Predictive Factors of Asymptomatic Spondylotic Cervical Spinal Stenosis in Patients with Symptomatic Lumbar Spinal Stenosis. World Neurosurg. 2021 Jul;151:e1051-e1058. doi: 10.1016/j.wneu.2021.05.054. Epub 2021 May 24. PMID: 34033955.
48. Jaumard NV, Udupa JK, Welch WC, Winkelstein BA. Kinematic magnetic resonance imaging to define the cervical facet joint space for the spine in neutral and torsion. Spine (Phila Pa 1976). 2014 Apr 15;39(8):664-72. doi: 10.1097/BRS.0000000000000206. PMID: 24430715.
49. Kadanka Z Jr, Adamova B, Kerkovsky M, Kadanka Z, Dusek L, Jurova B, Vlckova E, Bednarik J. Predictors of symptomatic myelopathy in degenerative cervical spinal cord compression. Brain Behav. 2017 Aug 11;7(9):e00797. doi: 10.1002/brb3.797. PMID: 28948090; PMCID: PMC5607559.
50. Kelly JC, Groarke PJ, Butler JS, Poynton AR, O'Byrne JM. The natural history and clinical syndromes of degenerative cervical spondylosis. Adv Orthop. 2012;2012:393642. doi: 10.1155/2012/393642. Epub 2011 Nov 28. PMID: 22162812; PMCID: PMC3227226.
51. Kalsi-Ryan S, Karadimas SK, Fehlings MG. Cervical spondylotic myelopathy: the clinical phenomenon and the current pathobiology of an increasingly prevalent and devastating disorder. Neuroscientist. 2013 Aug;19(4):409-21. doi: 10.1177/1073858412467377. Epub 2012 Nov 30. PMID: 23204243.
52. Ghogawala Z, Whitmore RG. Asymptomatic cervical canal stenosis: is there a risk of spinal cord injury? Spine J. 2013 Jun;13(6):613-4. doi: 10.1016/j.spinee.2013.04.001. PMID: 23747194.
53. Fakhoury J, Dowling TJ. Cervical Degenerative Disc Disease. 2023 Aug 14. In: StatPearls [Internet]. Treasure Island (FL): StatPearls Publishing; 2024 Jan–. PMID: 32809607.
54. Valošek J, Labounek R, Horák T, Horáková M, Bednařík P, Keřkovský M, Kočica J, Rohan T, Lenglet C, Cohen-Adad J, Hluštík P, Vlčková E, Kadaňka Z Jr, Bednařík J, Svatkova A. Diffusion magnetic resonance imaging reveals tract-specific microstructural correlates of electrophysiological impairments in non-myelopathic and myelopathic spinal cord compression. Eur J Neurol. 2021 Nov;28(11):3784-3797. doi: 10.1111/ene.15027. Epub 2021 Aug 4. Erratum in: Eur J Neurol. 2022 Aug;29(8):2551. PMID: 34288268; PMCID: PMC8530898.
55. Lee TH, Kim SJ, Lim SM. Prevalence of disc degeneration in asymptomatic korean subjects. Part 2 : cervical spine. J Korean Neurosurg Soc. 2013 Feb;53(2):89-95. doi: 10.3340/jkns.2013.53.2.89. Epub 2013 Feb 28. PMID: 23560172; PMCID: PMC3611065.

56. Liu B, Wu B, Van Hoof T, Okito JP, Liu Z, Zeng Z. Are the standard parameters of cervical spine alignment and range of motion related to age, sex, and cervical disc degeneration? J Neurosurg Spine. 2015 Sep;23(3):274-9. doi: 10.3171/2015.1.SPINE14489. Epub 2015 Jun 19. PMID: 26091436.
57. Machino M, Ando K, Kobayashi K, Morozumi M, Tanaka S, Ito K, Kato F, Ishiguro N, Imagama S. Cut off value in each gender and decade of 10-s grip and release and 10-s step test: A comparative study between 454 patients with cervical spondylotic myelopathy and 818 healthy subjects. Clin Neurol Neurosurg. 2019 Sep;184:105414. doi: 10.1016/j.clineuro.2019.105414. Epub 2019 Jul 5. PMID: 31306894.
58. Machino M, Ito K, Ando K, Kobayashi K, Nakashima H, Kato F, Imagama S. Normative Magnetic Resonance Imaging Data of Age-Related Degenerative Changes in Cervical Disc Morphology. World Neurosurg. 2021 Aug;152:e502-e511. doi: 10.1016/j.wneu.2021.05.123. Epub 2021 Jun 16. PMID: 34098133.
59. Machino M, Yukawa Y, Imagama S, Ito K, Katayama Y, Matsumoto T, Inoue T, Ouchida J, Tomita K, Ishiguro N, Kato F. Age-Related and Degenerative Changes in the Osseous Anatomy, Alignment, and Range of Motion of the Cervical Spine: A Comparative Study of Radiographic Data From 1016 Patients With Cervical Spondylotic Myelopathy and 1230 Asymptomatic Subjects. Spine (Phila Pa 1976). 2016 Mar;41(6):476-82. doi: 10.1097/BRS.0000000000001237. PMID: 26571180.
60. Martin AR, De Leener B, Cohen-Adad J, et alCan microstructural MRI detect subclinical tissue injury in subjects with asymptomatic cervical spinal cord compression? A prospective cohort studyBMJ Open 2018;8:e019809. doi: 10.1136/bmjopen-2017-019809
61. Horak T, Horakova M, Svatkova A, Kadanka Z, Kudlicka P, Valosek J, Rohan T, Kerkovsky M, Vlckova E, Kadanka Z, Deelchand DK, Henry PG, Bednarik J, Bednarik P. In vivo Molecular Signatures of Cervical Spinal Cord Pathology in Degenerative Compression. J Neurotrauma. 2021 Nov 1;38(21):2999-3010. doi: 10.1089/neu.2021.0151. PMID: 34428934; PMCID: PMC8917902.
62. Badhiwala JH, Wilson JR. The Natural History of Degenerative Cervical Myelopathy. Neurosurgery Clinics of North America. 2018;29(1):21-32. doi:10.1016/j.nec.2017.09.002
63. Banerjee A, Mowforth OD, Nouri A, et al. The Prevalence of Degenerative Cervical Myelopathy-Related Pathologies on Magnetic Resonance Imaging in Healthy/Asymptomatic Individuals: A Meta-Analysis of Published Studies and Comparison to a Symptomatic Cohort. Journal of Clinical Neuroscience. 2022;99:53-61. doi:10.1016/j.jocn.2022.03.002
64. Boody BS, Lendner M, Vaccaro AR. Ossification of the posterior longitudinal ligament in the cervical spine: a review. International Orthopaedics (SICOT). 2019;43(4):797-805. doi:10.1007/s00264-018-4106-5
65. Boody BS, Schroeder GD, Segar AH, Kepler CK. Should Asymptomatic Patients With Cervical Spinal Cord Compression and Spinal Cord Signal Change Undergo Surgical Intervention? Clin Spine Surg. 2019;32(3):87-90. doi:10.1097/BSD.0000000000000679
66. Brannigan JFM, Davies BM, Mowforth OD, et al. Management of mild degenerative cervical myelopathy and asymptomatic spinal cord compression: an international survey. Spinal Cord. Published online December 21, 2023. doi:10.1038/s41393-023-00945-8

67. Clarke H, Wood L, Eveleigh C. Developing a pathway for the management of patients presenting with suspected myelopathy in secondary care. Physiotherapy. 2022;114:e212-e213. doi:10.1016/j.physio.2021.12.197
68. Davies BM, Banerjee A, Mowforth OD, Kotter MRN, Newcombe VFJ. Is the type and/or co-existence of degenerative spinal pathology associated with the occurrence of degenerative cervical myelopathy? A single centre retrospective analysis of individuals with MRI defined cervical cord compression. Journal of Clinical Neuroscience. 2023;117:84-90. doi:10.1016/j.jocn.2023.09.015
69. Donnally CJ, Patel PD, Canseco JA, Vaccaro AR, Kepler CK. Current Management of Cervical Spondylotic Myelopathy. Clinical Spine Surgery: A Spine Publication. 2022;35(1):E68-E76. doi:10.1097/BSD.0000000000001113
70. Donnally CJ, Patel PD, Canseco JA, Vaccaro AR, Kepler CK. Current Management of Cervical Spondylotic Myelopathy. Clin Spine Surg. 2022;35(1):E68-E76. doi:10.1097/BSD.0000000000001113
71. Dowlati E, Mualem W, Black J, et al. Should asymptomatic cervical stenosis be treated in the setting of progressive thoracic myelopathy? A systematic review of the literature. Eur Spine J. 2022;31(2):275-287. doi:10.1007/s00586-021-07046-1
72. Fakhoury J, Dowling TJ. Cervical Degenerative Disc Disease. In: StatPearls. StatPearls Publishing; 2024. Accessed October 19, 2024. http://www.ncbi.nlm.nih.gov/books/NBK560772/
73. Farahbakhsh F, Khosravi S, Baigi V, et al. The Prevalence of Asymptomatic Cervical Spinal Cord Compression in Individuals Presenting With Symptomatic Lumbar Spinal Stenosis: A Meta-Analysis. Global Spine Journal. 2024;14(3):1052-1060. doi:10.1177/21925682231202776
74. Gallagher DO, Taghlabi KM, Bondar K, Saifi C. Degenerative Cervical Myelopathy: A Concept Review and Clinical Approach. Clinical Spine Surgery. 2024;37(1):1. doi:10.1097/BSD.0000000000001463
75. Grodzinski B, Stubbs DJ, Davies BM. Most degenerative cervical myelopathy remains undiagnosed, particularly amongst the elderly: modelling the prevalence of degenerative cervical myelopathy in the United Kingdom. J Neurol. 2023;270(1):311-319. doi:10.1007/s00415-022-11349-8
76. Hameed S, Muhammad F, Haynes G, Smith L, Khan AF, Smith ZA. Early neurological changes in aging cervical spine: insights from PROMIS mobility assessment. GeroScience. 2024;46(3):3123-3134. doi:10.1007/s11357-023-01050-7
77. Hejrati N, Moghaddamjou A, Marathe N, Fehlings MG. Degenerative Cervical Myelopathy: Towards a Personalized Approach. Can J Neurol Sci. 2022;49(6):729-740. doi:10.1017/cjn.2021.214
78. Horak T, Horakova M, Svatkova A, et al. In vivo Molecular Signatures of Cervical Spinal Cord Pathology in Degenerative Compression. Journal of Neurotrauma. 2021;38(21):2999-3010. doi:10.1089/neu.2021.0151
79. Houten JK, Shahsavarani S, Verma RB. The Natural History of Degenerative Cervical Myelopathy. 2022;35(10).
80. Jay Gaffney C, Ryan Spiker W. Treating multilevel (three or four level) cervical myelopathy with ACDF or ACCF. Seminars in Spine Surgery. 2014;26(3):122-127. doi:10.1053/j.semss.2014.08.002
81. Kane SF, Abadie KV, Willson A. Degenerative Cervical Myelopathy: Recognition and Management. 2020;102(12).

82. Kerkovský M, Bednarík J, Dušek L, et al. Magnetic Resonance Diffusion Tensor Imaging in Patients With Cervical Spondylotic Spinal Cord Compression: Correlations Between Clinical and Electrophysiological Findings. Spine. 2012;37(1):48-56. doi:10.1097/BRS.0b013e31820e6c35
83. Kim MW, Kang CN, Choi SH. Update of the Natural History, Pathophysiology, and Treatment Strategies of Degenerative Cervical Myelopathy: A Narrative Review. Asian Spine J. 2023;17(1):213-221. doi:10.31616/asj.2022.0440
84. Le HV, Wick JB, Van BW, Klineberg EO. Ossification of the Posterior Longitudinal Ligament: Pathophysiology, Diagnosis, and Management. J Am Acad Orthop Surg. Published online May 18, 2022. doi:10.5435/JAAOS-D-22-00049
85. Maus TP. Imaging of Spinal Stenosis. Radiologic Clinics of North America. 2012;50(4):651-679. doi:10.1016/j.rcl.2012.04.007
86. McCormick JR, Sama AJ, Schiller NC, Butler AJ, Donnally CJ. Cervical Spondylotic Myelopathy: A Guide to Diagnosis and Management. J Am Board Fam Med. 2020;33(2):303-313. doi:10.3122/jabfm.2020.02.190195
87. Nakashima H, Yukawa Y, Suda K, Yamagata M, Ueta T, Kato F. Narrow cervical canal in 1211 asymptomatic healthy subjects: the relationship with spinal cord compression on MRI. Eur Spine J. 2016;25(7):2149-2154. doi:10.1007/s00586-016-4608-z
88. Nouri A, Tessitore E, Molliqaj G, et al. Degenerative Cervical Myelopathy: Development and Natural History [AO Spine RECODE-DCM Research Priority Number 2]. Global Spine Journal. 2022;12(1_suppl):39S-54S. doi:10.1177/21925682211036071
89. Oei MW, Evens AL, Bhatt AA, Garner HW. Imaging of the Aging Spine. Radiologic Clinics of North America. 2022;60(4):629-640. doi:10.1016/j.rcl.2022.03.006
90. Onofrei LV, Henrie AM. Cervical and Thoracic Spondylotic Myelopathies. Semin Neurol. 2021;41(03):239-246. doi:10.1055/s-0041-1725144
91. Rodrigues-Pinto R, Montenegro TS, Davies BM, et al. Optimizing the Application of Surgery for Degenerative Cervical Myelopathy [AO Spine RECODE-DCM Research Priority Number 10]. Global Spine Journal. 2022;12(1_suppl):147S-158S. doi:10.1177/21925682211062494
92. Sarraj M, Hache P, Foroutan F, et al. Natural history of degenerative cervical myelopathy: a meta-analysis and neurologic deterioration survival curve synthesis. The Spine Journal. 2024;24(1):46-56. doi:10.1016/j.spinee.2023.07.020
93. Valošek J, Bednařík P, Keřkovský M, Hluštík P, Bednařík J, Svatkova A. Quantitative MR Markers in Non-Myelopathic Spinal Cord Compression: A Narrative Review. JCM. 2022;11(9):2301. doi:10.3390/jcm11092301
94. Wang C, Ellingson BM, Oughourlian TC, Salamon N, Holly LT. Evolution of brain functional plasticity associated with increasing symptom severity in degenerative cervical myelopathy. eBioMedicine. 2022;84:104255. doi:10.1016/j.ebiom.2022.104255
95. Williams J, D’Amore P, Redlich N, et al. Degenerative Cervical Myelopathy. Orthopedic Clinics of North America. 2022;53(4):509-521. doi:10.1016/j.ocl.2022.05.007
96. Wilson JR, Barry S, Fischer DJ, et al. Frequency, timing, and predictors of neurological dysfunction in the nonmyelopathic patient with cervical spinal cord compression, canal stenosis, and/or ossification of

the posterior longitudinal ligament. Spine (Phila Pa 1976). 2013;38(22 Suppl 1):S37-54. doi:10.1097/BRS.0b013e3182a7f2e7
